# A Genetic Variant in Long Non-Coding RNA *HULC* Contributes to Risk of HBV-Related Hepatocellular Carcinoma in a Chinese Population

**DOI:** 10.1371/journal.pone.0035145

**Published:** 2012-04-06

**Authors:** Yao Liu, Shandong Pan, Li Liu, Xiangjun Zhai, Jibin Liu, Juan Wen, Yixin Zhang, Jianguo Chen, Hongbing Shen, Zhibin Hu

**Affiliations:** 1 Department of Epidemiology and biostatistics, MOE Key Laboratory of Modern Toxicology, School of Public Health, Nanjing Medical University, Nanjing, China; 2 Department of Hepatobiliary Surgery, Nantong Tumor Hospital, Nantong, China; 3 Institute of Digestive Endoscopy and Medical Center for Digestive Diseases, Second Affiliated Hospital of Nanjing Medical University, Nanjing, China; 4 Department of Infection Diseases, Jiangsu Province Center for Disease Prevention and Control, Nanjing, China; 5 Qidong Liver Cancer Research Institute, Qidong, China; 6 Section of Clinical Epidemiology, Jiangsu Key Lab of Cancer Biomarkers, Prevention and Treatment, Cancer Center, Nanjing Medical University, Nanjing, China; 7 State Key Laboratory of Reproductive Medicine, Nanjing Medical University, Nanjing, China; IPO, Inst Port Oncology, Portugal

## Abstract

**Background:**

Recently, several studies have demonstrated that two long non-coding RNAs (lncRNAs), *HULC* and *MALAT1*, may participate in hepatocellular carcinoma (HCC) development and progression. However, genetic variations in the two lncRNAs and their associations with HCC susceptibility have not been reported. In this study, we hypothesized that single nucleotide polymorphisms (SNPs) in *HULC* and *MALAT1* may contribute to HCC risk.

**Methods:**

We conducted a case-control study and genotyped two SNPs, rs7763881 in *HULC* and rs619586 in *MALAT1*, in 1300 HBV positive HCC patients, 1344 HBV persistent carriers and 1344 subjects with HBV natural clearance to test the associations between the two SNPs and susceptibility to HCC and HBV chronic infection.

**Results:**

The variant genotypes of rs7763881 were significantly associated with decreased HCC risk in a dominant genetic model [AC/CC *vs*. AA: adjusted odds ration (OR)  =  0.81, 95% confidence intervals (CIs)  =  0.68–0.97, *P*  =  0.022]. Furthermore, the variant genotypes of rs619586 was associated with decreased HCC risk with a borderline significance (AG/GG *vs*. AA: adjusted OR  =  0.81, 95% CIs  =  0.65–1.01, *P*  =  0.057). However, no significant association was found between the two SNPs and HBV clearance.

**Conclusions:**

The variant genotypes of rs7763881 in *HULC* may contribute to decreased susceptibility to HCC in HBV persistent carriers.

## Introduction

Hepatocellular carcinoma (HCC) is the fifth most common cancer in men and the seventh in women in the world, and the new cases of HCC in developing countries accounted for almost 85% of all patients [1]. The major risk factors for HCC include chronic infections with the hepatitis B or C viruses, alcohol consumption, and foodstuff contamination with aflatoxins [2]–[5]. Among those, the hepatitis B virus (HBV) infection is of particular interest, for its coherent distribution with the HCC prevalence [6]. In China, although national vaccination of HBV has been carried out for years, the infection rate of HBV is still at a high level, approximately 5–8% in the general population [7], [8]. However, only a small fraction of HBV persistent carriers developed HCC, suggesting genetic variation may play roles in the carcinogenesis of HCC after HBV infection. Besides, HBV persistent infection or HBV natural clearance may also be influenced by complex factors of viral, host age, environmental and genetic makeups.

Based on the latest knowledge, RNA is no longer only the bridge between DNA and protein [9]. In recent years, more and more non-coding RNAs have been identified, including short non-coding RNAs(microRNAs) and long non-coding RNAs(lncRNAs). Guttman *et al* found over a thousand highly conserved lncRNAs in mammals through chromatin signature mapping [10]. However, few publications focused on the origin and biological functions of lncRNAs than those of microRNAs. Recently, several studies reported that lncRNAs were dysregulated in different caners [11]–[13], though their specific roles in caner development and progression were largely unknown.

*HULC*, short for Highly Up-regulated in Liver Cancer, is about 1.6 k nucleotide long, containing two exons but not translated [13]. It has been identified that *HULC* is highly upregulated in HCC and colorectal cancer that metastasized to livers [13], [14]. *MALAT1*, abbreviation of Metastasis-Associated Lung Adenocarcinoma Transcript 1, is about 8k nucleotide long and has been demonstrated to be dysregulated in a variety of cancers including HCC [11], [12].

Genetic variations in lncRNAs and their associations with cancer susceptibility have rarely been reported. Recently, Jin G *et al* reported the association between polymorphisms in lncRNAs and prostate cancer [15]. In this study, we hypothesized that single nucleotide polymorphisms (SNPs) in *HULC* and *MALAT1* may contribute to risk of HBV chronic infection and HCC. To test our hypothesis, we conducted a case-control study of 1300 HBV-positive HCC patients, 1344 HBV persistent carriers and 1344 subjects with HBV natural clearance to assess the associations between SNPs in *HULC* and *MALAT1* and the susceptibility to HBV chronic infection and HCC.

## Methods

### Study Subjects

This study was approved by the institutional review board of Nanjing Medical University. The subjects’ enrollment was described previously [16]. The newly diagnosed HCC patients were consecutively recruited between January 2006 and December 2010 from the First Affiliated Hospital of Nanjing Medical University, the Nantong Tumor Hospital, and the Qidong liver cancer institute of Jiangsu Province, China. Each patient was confirmed by a pathological examination and/or α-fetoprotein elevation (>400 ng/ml) combined with imaging examination (Magnetic resonance imaging, MRI and/or computerized tomography, CT). Eventually, 1300 HBV positive (HCV negative) HCC cases were included.

The controls were screened for the HBV/HCV markers from two cities in Jiangsu Province (9720 persons from Changzhou and 48422 persons from Zhangjiagang) in 2004 and 2009, respectively. Two groups of controls were used in the current study, one is the HBV persistent carriers and the other is persons with HBV natural clearance. HBV persistent carriers were positive for both HBV surface antigen (HBsAg) and antibody to hepatitis B core antigen (anti-HBc), negative for HCV antibody (anti-HCV). Subjects with HBV natural clearance were negative for HBsAg and anti-HCV, plus positive for both antibody to hepatitis B surface antigen (anti-HBs) and anti-HBc. These people declared no hepatitis B vaccination history. About 865 (8.9%) HBV persistent carriers and 1759 (18.1%) subjects with HBV natural clearance were identified from Changzhou; while 2156 (4.5%) HBV persistent carriers and 7851 (16.2%) subjects with HBV natural clearance were identified from Zhangjiagang. Then, we randomly selected 1344 HBV persistent carriers and 1344 HBV natural clearance people from the two cities, which were matched to the HCC cases on age and sex. These selected controls declared no previous malignancy.

All participants were unrelated ethnic Han Chinese. After written informed consent was obtained, each participant was surveyed by a structured questionnaire to collect demographic and exposure information. Individuals who smoked one cigarette per day for over one year were defined as smokers, and those who consumed one or more alcohol drinks a week for over six months were categorized as alcohol drinkers. After the interview, about 5 milliliter venous blood sample was collected from each participant.

### Serological Testing

HBsAg, anti-HBs, anti-HBc and anti-HCV were detected from each participant’s serum by the enzyme-linked immunosorbent assay (Kehua Bio-engineering Co., Ltd., Shanghai, China) following the manufacturer’s instructions as described previously [16].

### SNPs Selection and Genotyping

Based on the data from Hapmap database and the criteria of minor allele frequency (MAF) > 0.05 in Han Chinese, we found five common SNPs (rs1328868, rs2038540, rs1328867, rs7763881 and rs1328866) in *HULC*, all of which are in high linkage disequilibrium (LD). Thus, we genotyped only one SNP, rs7763881. Similarly, there are five common SNPs (rs11227209, rs619586, rs7927113, rs664589 and rs3200401) in *MALAT1*, all of which are in one LD block and we chose rs619586 as the tagging SNP.

Genomic DNA was extracted from a leukocyte pellet by traditional proteinase K digestion and followed by phenol-chloroform extraction and ethanol precipitation. SNPs rs7763881 A>C and rs619586 A>G were genotyped using the TaqMan allelic discrimination assay on a 7900 system (Applied Biosystems Inc.). The primers and probes for rs7763881 were as follows: Primer: sense, 5'-GGATAAAGGAATTCTGGGAAATGTAG-3', antisense, 5'-GGTGCTGTGTTGTGGATTTGC-3'; Probe: allele A, FAM-TTTGTCTGAATTGACCTAT-MGB, allele C, HEX-TTGTCTGACTTGACCTAT-MGB. The primers and probes for rs619586 were as follows: Primer: sense, 5'-AAAGCCCTGAACTATCACACTTTAATC-3', antisense, 5'-CACAAAACCCCCGGAACTT-3'; Probe: allele A, FAM-ACTATACCTACTGTCCC-MGB, allele G, HEX-CTATACCTGCTGTCCC-MGB. The genotyping was performed blindly without knowing the subjects’ case or control status. Two blank (water) controls in each 384-well plate were used for quality control and more than 10% samples were randomly selected to repeat, yielding a 100% concordance rate.

### Statistical Analysis

Differences of demographic characteristics and genotype frequencies of the two SNPs between the cases and controls were calculated by the Student’s t-test (for continuous variables) and χ^2^ test (for categorical variables). Associations between the genotypes and risk of HCC and HBV chronic infection were estimated by computing odds ratios (OR) and their 95% confidence intervals (CIs) from logistic regression analyses. Heterogeneity of associations between subgroups was assessed by the χ^2^ -based Q test. All of the statistical analyses were performed with R software (version 2.13.0; The R Foundation for Statistical Computing). All tests were two-sided and the criterion of statistical significance was set at *P* < 0.05.

## Results

The demographic characteristics of the 1300 HBV positive HCC patients, 1344 HBV persistent carriers and 1344 subjects with HBV nature clearance were summarized previously [16]. There were no significant differences in the distribution of age, sex and smoking rate between the three groups. However, the drinking rate was significantly higher in HCC cases than that in controls ( both *P* < 0.001 for HCC cases compare to HBV persistent carriers and clearance controls).

The genotyping call rates were 98.2% for rs7763881 and 98.5% for rs619589 and the observed genotype frequencies among the controls were in HWE (*P*  =  0.785 and 0.453 for rs7763881 and rs619586, respectively). The genotype distributions of the two variants in HBV positive HCC cases, HBV persistent carriers and HBV natural clearance subjects are shown in Table 1. Compared with individuals carrying wild-type AA genotype of rs7763881, those with AC and CC genotypes had a decreased HCC risk with adjusted ORs of 0.79 (95% CIs  =  0.66–0.95) and 0.87 (95% CIs  =  0.70–1.09), respectively. The variant AC/CC genotypes of rs7763881 significantly decreased HCC risk by 19% (Adjusted OR  =  0.81, 95% CIs  =  0.68–0.97, *P*  =  0.022). Similarly, variant genotypes of rs6682925 were associated with non-significant decreased HCC risk compared with the wild-type AA genotype (adjusted OR  =  0.82, 95% CIs  =  0.66–1.03 for AG; adjusted OR =  0.50, 95% CIs = 0.17–1.50 for GG). The variant genotypes AG/GG of rs619586 was associated with a decreased HCC risk with a borderline significance (adjusted OR  =  0.81, 95%CIs  =  0.65–1.01, *P*  =  0.057). However, no significant association was found between the two SNPs and HBV clearance.

**Table 1:** Distribution of Alleles and Genotypes of two SNPs and Their Association with HCC Risk and HBV Chronic infection.

SNPs	HCC Case (n = 1300)		HBV persistent carriers (n = 1344)		HBV natural clearance (n = 1344)		Adjusted OR^a^	*P*	Adjusted OR b	*P*
rs7763881	1271		1316		1327					
AA	371	29.2	333	25.3	367	27.7	1.00		1.00	
AC	617	48.5	695	52.8	633	47.7	0.79(0.66–0.95)		1.20(1.00–1.45)	
CC	283	22.3	288	21.9	327	24.6	0.87(0.70–1.09)		0.96(0.78–1.20)	
AC/CC	900	70.8	983	74.7	960	72.3	**0.81(0.68–0.97)**	**0.022**	1.12(0.94–1.34)	0.189
rs619586	1268		1330		1330					
AA	1094	86.3	1115	83.8	1116	83.9	1.00		1.00	
AG	169	13.3	205	15.4	202	15.2	0.82(0.66–1.03)		1.01(0.82–1.25)	
GG	5	0.4	10	0.8	12	0.9	0.50(0.17–1.50)		0.83(0.36–1.94)	
AG/GG	174	13.7	215	16.2	214	16.1	0.81(0.65–1.01)	0.057	1.00(0.81–1.23)	0.989

NOTE: Logistic regression analyses adjusted for age, sex, smoking status and drinking status.

a HCC patients *vs.* HBV persistent carriers.

b HBV persistent carriers *vs.* HBV natural clearance subjects.

Stratified analyses of rs7763881 and rs619586 are summarized in Table S1. The protective effect for rs7763881 was more prominent in subjects older than 53 years old (adjusted OR  =  0.66, 95% CIs  =  0.51–0.85, *P* for heterogeneity test  =  0.036). For rs619586, a significant protective effect was only observed for never drinkers (adjusted OR  =  0.66, 95% CIs  =  0.47–0.93, *P*  =  0.015).

## Discussion

To the best of our knowledge, this is the first study that has provided evidence that common SNPs in lncRNAs might be associated with HCC susceptibility. We identified rs7763881 in *HULC* was significantly associated with HCC susceptibility in HBV persistent carriers, while rs619586 was protective for non-drinkers’ HCC risk in subgroup analysis.

Panzitt *et al* firstly reported that *HULC* was highly up-regulated in HCC as a novel non-coding RNA [13]. In addition, siRNA-mediated knockdown of *HULC* in 2 HCC cell lines resulted in altered expression of several genes, some of which were known to be affected in HCC, including cyclin-dependent kinase 8 and MAP kinase interacting serine/threonine kinase 2 [13]. Furthermore, the expression of *HULC* was extremely tissue-specific. Panzitt *et al* reported that *HULC* was not detected in tissues other than liver or their associated neoplasms [13]. Matouk *et al* reported that *HULC* was detected in colorectal carcinomas that metastasize to livers, but not the primary colorectal carcinomas samples nor their normal counterparts [13], [14].

Based on the Hapmap database, there are five SNPs in *HULC*, all of which are in high LD. Among those, rs7763881 was in complete LD with rs1328867 (r-square  =  1), which is located in the promoter region of *HULC*. According to data from UCSC (http://genome.ucsc.edu) and TFSEARCH 1.3, the wild-type allele T of rs1328867 is predicted to bind several transcription factors including C-Myc, while the variant allele C is not. C-Myc plays critical roles in regulating cellular growth, differentiation and apoptosis in both normal and neoplastic liver cells [17]. In our study, with a relative large sample size and a power of 76%, we found that the variant allele C of rs7763881 was associated with a decreased HCC risk, which was consistent with the biological relevance of *HULC*.

*MALAT1* is highly conserved within the mammalian lineage [18]. It is enriched in nuclear speckles in inter-phase cells and concentrates in mitotic interchromatin granule clusters [19]. It is co-localized with pre-mRNA-splicing factor SF2/ASF and CC3 antigen in the nuclear speckles [20]. *MALAT1* was found rearranged in renal tumors harboring the t(6;11)(p21;q13) translocation and in a liver mesenchymal hamartoma harboring the t(11;19)(q11;q13.4) translocation [21], [22]. In this study, we found that *MALAT1* rs619586 was associated with a decreased HCC risk with a borderline significance and was significantly protective for HCC risk in never drinkers. The lack of significance in the overall population might be due to the modest association and the relative low MAF of the SNP (MAF  =  0.11), and we have only about 40% power to detect the effect in this population with our current sample size. Larger studies are warranted to clarify the associations between rs619586 in *MALAT1* and HCC risk.

In conclusion, the variant genotypes of rs7763881 in *HULC* may contribute to decreased HCC susceptibility in HBV persistent carriers. Association studies with diverse population and further functional studies of the variants are warranted to confirm our findings.

## Supporting Information

Table S1 **Stratified Analysis between the two SNPs and HCC Risk. **NOTE: Logistic regression analyses adjusted for age, sex, smoking status and drinking status in dominant genetic model (excluded the stratified factor in each stratum).
^a^ HCC patients *vs.* HBV persistent carriers.
^b^ *P* for heterogeneity****.
(DOC)
