# Supplementary material for: A Genetic Variant in Long Non-Coding RNA HULC Contributes to Risk of HBV-Related Hepatocellular Carcinoma in a Chinese Population
Source: PLoS One. 2012 Apr 6;7(4):e35145. doi: 10.1371/journal.pone.0035145 (PMC3320879; doi:10.1371/journal.pone.0035145)
Supplement: Table S1 — Stratified Analysis between the two SNPs and HCC Risk. NOTE: Logistic regression analyses adjusted for age, sex, smoking status and drinking status in dominant genetic model (excluded the stratified factor in each stratum). a HCC patients vs. HBV persistent carriers. b P for heterogeneity. (DOC) [file pone.0035145.s001.doc]

Table S1. Stratified Analysis between the two SNPs and HCC Risk

| Characteristics | rs7763881 | | | | | | rs619586 | | | | | |
| --- | --- | --- | --- | --- | --- | --- | --- | --- | --- | --- | --- | --- |
|  | HCC Case (n=1300) | | HBV persistent carriers (n=1344) | | OR (95%CIs) ^a^ | *P* ^b^ | HCC Case (n=1300) | | HBV persistent carriers (n=1344) | | OR (95%CIs) ^a^ | *P* ^b^ |
|  |  |  |  |  |  |  |  |  |  |  |  |  |
|  | AA | AC/CC | AA | AC/CC |  |  | AA | AG/GG | AA | AG/GG |  |  |
| Age (year) |  | | | |  |  |  | | | |  |  |
| ≤53 | 189(27.9) | 489(72.1) | 187(27.2) | 501(72.8) | 0.96(0.76-1.23) | **0.036** | 590(87.3) | 86(12.7) | 587(84.5) | 108(15.5) | 0.79(0.58-1.07) | 0.704 |
| >53 | 182(30.7) | 411(69.3) | 146(23.2) | 482(76.8) | **0.66(0.51-0.85)** |  | 504(85.1) | 88(14.9) | 528(83.1) | 107(16.9) | 0.84(0.61-1.15) |  |
| Gender |  |  |  |  |  |  |  |  |  |  |  |  |
| Male | 311(28.8) | 768(71.2) | 274(24.6) | 841(75.4) | **0.80(0.66-0.96)** | 0.748 | 926(85.9) | 152(14.1) | 946(84.1) | 179(15.9) | 0.84(0.66-1.07) | 0.275 |
| Female | 60(31.2) | 132(68.8) | 59(29.4) | 142(70.6) | 0.87(0.54-1.40) |  | 168(88.4) | 22(11.6) | 169(82.4) | 36(17.6) | 0.80(0.43-1.50) |  |
| Smoking status |  |  |  |  |  |  |  |  |  |  |  |  |
| Never | 161(31.3) | 354(68.7) | 145(25.9) | 415(74.1) | 0.75(0.58-0.98) | 0.673 | 439(86.4) | 69(13.6) | 470(82.7) | 98(17.3) | 0.77(0.55-1.08) | 0.478 |
| Ever | 210(27.8) | 546(72.2) | 188(24.9) | 568(75.1) | **0.81(0.64-1.04)** |  | 655(86.2) | 105(13.8) | 645(84.6) | 117(15.4) | 0.85(0.63-1.15) |  |
| Drinking status |  |  |  |  |  |  |  |  |  |  |  |  |
| Never | 157(29.8) | 370(70.2) | 195(26.7) | 534(73.3) | 0.85(0.66-1.10) | 0.498 | 458(88.1) | 62(11.9) | 615(83.4) | 122(16.6) | **0.66(0.47-0.93)** | 0.149 |
| Ever | 214(28.8) | 530(71.2) | 138(23.5) | 449(76.5) | **0.75(0.58-0.97)** |  | 636(85.0) | 112(15.0) | 500(84.3) | 93(15.7) | 0.95(0.70-1.29) |  |
